# Systems biology approaches toward autosomal dominant polycystic kidney disease (ADPKD)

**DOI:** 10.1186/s40169-019-0254-5

**Published:** 2020-01-06

**Authors:** Ilnaz Rahimmanesh, Razieh Fatehi

**Affiliations:** Department of Genetics and Molecular Biology, School of Medicine, Isfahan University of Medical Sciences, Isfahan, Iran

**Keywords:** Autosomal dominant polycystic kidney disease, Microarray, Protein interaction network, Signal pathway

## Abstract

**Background:**

Autosomal dominant polycystic kidney disease (ADPKD), a common of monogenetic disorder caused by the polycystic kidney disease-1 (PKD1) or PKD2 genes deficiency. In this study, we have re-analyzed a microarray dataset to generate a holistic view of this disease.

**Methodology:**

GSE7869, an expression profiling dataset was downloaded from the Gene Expression Omnibus (GEO) database. After quality control assessment, using GEO2R tool of GEO, genes with adjusted p-value ≤ 0.05 were determined as differentially expressed (DE). The expression profiles from ADPKD samples in different sizes were compared. Using CluePedia plugin of Cytoscape software, the protein–protein interaction (PPI) networks were constructed and analyzed by Cytoscape NetworkAnalyzer tool and MCODE application. Pathway enrichment analysis of clustered genes by MCODE with the high centrality parameters in PPI networks was performed using Cytoscape ClueGO plugin. Moreover, by Enrichr database, microRNAs (miRNAs) and transcription factors (TFs) targeted DE genes were identified.

**Results:**

In this study to explore the molecular pathogenesis of kidney in ADPKD, mRNA expression profiles of cysts from patients in different sizes were re-analyzed. The comparisons were performed between normal with minimally cystic tissue (MCT) samples, MCTs with small cysts, and small cysts with large cysts. 512, 7024, and 655 DE genes were determined, respectively. The top central genes, e.g. END1, EGFR, and FOXO1 were identified with topology and clustering analysis. DE genes that were significantly enriched in PPI networks are critical genes and their roles in ADPKD remain to be assessed in future experimental studies beside miRNAs and TFs predicted. Furthermore, the functional analysis resulted in which most of them are expected to be associated with ADPKD pathogenesis, such as signal pathways that involved in cell growth, inflammation, and cell polarity.

**Conclusion:**

We have here explored systematic approaches for molecular mechanisms assay of ADPKD as a monogenic disease, which may also be used for other monogenetic diseases beside complex diseases to provide suitable therapeutic targets.

## Background

The hereditary autosomal dominant polycystic kidney disease (ADPKD) is the most common monogenic disorder. ADPKD is a multi-systematic disease diagnosed by growing multiple cysts on kidneys. liver cysts and cerebral aneurysms are also the main clinical findings of disease [1]. ADPKD is genetically heterogeneous and results from mutations in at least two genes, Polycystic Kidney Disease-1 (PKD1) or PKD-2 [2]. These genes encode transmembrane proteins, Polycystin-1 (PC-1) and Polycystin-2 (PC-2) which form a functional complex [3]. This protein complex, similar to other proteins are affected in polycystic kidney diseases locate in primary cilia of epithelial and endothelial cells [4]. PC-1 known as a cell surface receptor and PC-2 is a cation channel and both of them play a critical role in controlling of signaling pathways related to proliferation, apoptosis, and cell polarities through Ca2+ homeostasis regulation [5]. In spite of numerous studies related to polycystins functions, their roles are poorly understood. Regarding this major limitation being sensible to recognize the underlying mechanisms, systems biology approaches with a holistic view of the molecular mechanisms of disorders, have the potential to overcome these limitations. These approaches with comprehensive interpretation, using high throughput data extracted from omics data, provide the opportunity to represent the behavior of networks and emerge new therapeutic strategies. Therefore, we re-analyzed the array dataset deposited by Song X et al. which was compared transcription profiling of all samples from PKD1 patients with normal tissue, and gene set enrichment analysis (GSEA) was performed [6]. But here, we have shown large-scale protein interaction networks. For deeply understanding of central genes that related with phenotypes of disease in each step, network and clustering analysis were carried out. These revealed some of the key genes, such as EDN1, EGFR, ARF6, FOXO1, and ITGB5 involved during disease. Pathways were identified with enrichment analysis with the notice on cysts size, from early to late steps. Moreover, for the purpose of assay the regulatory mechanisms of DE genes, microRNAs (miRNAs) and transcription factors (TFs) enriched with DE genes were predicted.

## Methods

### Microarray data and DE genes screening

Microarray dataset with accession number “GSE7869” from the Gene Expression Omnibus (GEO) database was extracted. The quality of transcriptomics dataset was measured by principal component analysis (PCA) through the ggplot2 package and prcomp function of R [7]. Using GEO2R a web tool of GEO, groups were compared to detect genes that are differentially expressed with cysts growth. Samples of normal tissues (n = 3), minimally cystic tissues (n = 5), small cysts (n = 5), and large cysts (n = 3) were compared based on during the time of disease progression, using Student’s t-test, respectively. Benjamini–Hochberg false discovery rate (FDR) was used for p-value correction. Genes were declared as differentially expressed, had an adjusted p-value less than 0.05.

### Protein-protein interaction networks construction

The protein–protein interaction (PPI) networks were built with DE genes. For networks construction, CluePedia plugin version 1.5.2 [8] of Cytoscape software version 3.7.1 [9] was used. STRING database with confidence cutoff 0.80 was provided, for retrieving interactions [10]. Networks topology was investigated using the NetworkAnalyzer tool of Cytoscape [11]. “Molecular Complex Detection” (MCOD) plugin of Cytoscape detected modules, highly connected sub-networks, based on default settings [12].

### Pathway enrichment analysis

Functional analysis of genes clustered with MCODE was done by Cytoscape ClueGO plugin version 2.5.2 [13]. Reactome [14] and KEGG (Kyoto Encyclopedia of Genes and Genomes) [15] databases were chosen for retrieving pathways. Bonferroni step down was applied for p-value correction, and signaling pathways with adjusted p-value ≤ 0.05 were determined.

### miRNA and TF enrichment analysis

The microRNAs (miRNAs) and transcription factors (TFs), key regulators of genes, were predicted by Enrichr web server [16]. TargetScan microRNA 2017 and ChEA 2016 libraries were used for miRNA and TF enrichment analysis, respectively. Adjusted p-value less than 0.05 was considered as the significant threshold. The miRNAs with more targeted genes were selected.

## Results

### By microarray data analysis, differentially expressed genes were identified

**Fig. 1:**
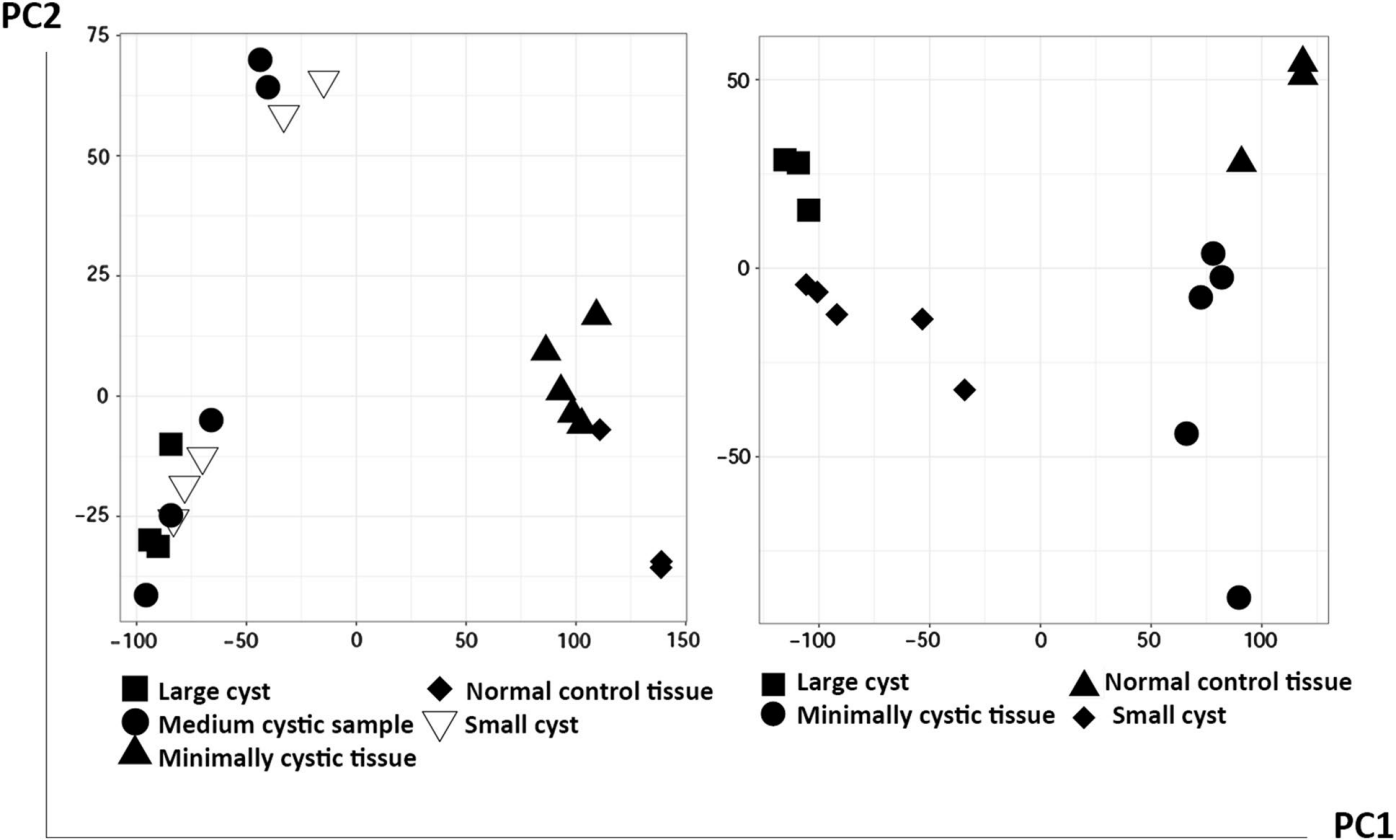
The quality of the microarray dataset is satisfying. The Principle component analysis results of the GSE7869 dataset were shown the samples were separated appropriately

**Fig. 2:**
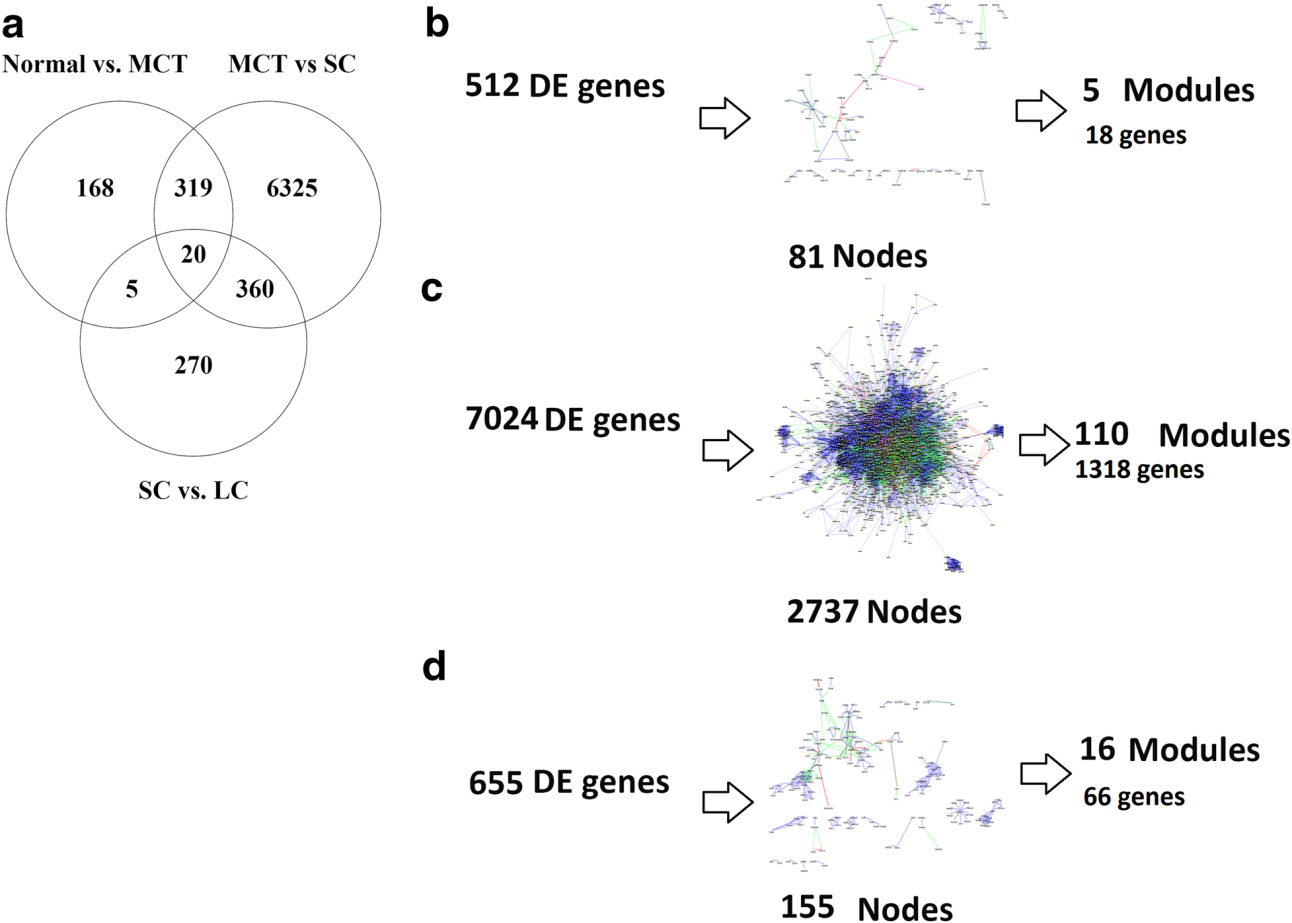
The overlapping of differentially expressed genes and protein–protein interaction networks. The protein- protein interaction networks were built with differentially expressed genes. **b**: Normal vs. MCT, **c**: MCT vs. SC, **d**: SC vs. LC)

The microarray dataset “GSE7869” which includes renal cysts in different sizes; small cysts (SC) less than 1 mm, medium cysts between 10 and 25 mm, and large cysts (LC) greater than 50 mm have been analyzed. Minimally cystic tissues (MCT) obtained from healthy parts of the renal cortex of PKD1 patients were considered as heterozygote samples. In quality assay step except medium cysts, the samples were segregated based on their states (normal tissue, minimally cystic tissue, small cyst, and large cyst), indicate the acceptable quality of this dataset (Fig. 1). Using GEO2R tool, we obtained 512, 7024, and 655 genes which are significantly variably expressed between normal vs. MCT samples, MCT vs. SC samples, and SC vs. LC samples, respectively (Additional file 1). Interestingly, these sets of DE genes have few overlapping genes (Fig. 2a).

### Protein–protein interaction networks were constructed

The PPI networks with DE genes were constructed. Links between genes were selected based on activation, binding, post-translational modification, and inhibition interactions. PPI networks are shown small cyst growth phase is an important and complex step during the progression of the disease. 81, 2737, and 155 nodes (genes) are in PPI networks (normal vs. MCT, MCT vs. SC, and SC vs. LC), respectively (Fig. 2b–d). The MCODE application identified protein clusters in networks. These protein complexes and modules are highly interconnected subnetworks with the most effective genes. Network topology were measured based on the graph theory concepts such as degree, betweenness, and closeness centrality. The seed gene with the highest centrality is EDN1 in the early stage, normal vs. MCT comparison. Seed genes such as EGFR, ARF6, WWTR, SMURF2, TGFB2, and HSD17B8 are critical genes in the comparison of MCT with SC. FOXO1, EDN1, and ITGB5 are introduced as central genes in the late stage, SC vs. LC comparison. Some of these genes including EGFR and EDN1 have been recognized related to ADPKD in previous experimental studies [17, 18] and other genes are candidates for future studies. The genes are represented in Table 1.

**Table 1:** Top clustered genes in the PPI networks. The seed genes with the highest density in PP networks are shown

GOID	Betweenness centrality	Closeness centrality	Degree	GOID	Betweenness centrality	Closeness centrality	Degree
Normal vs. MCT				HSF1	0.002468	0.285681	17
EDN1	0.412698	0.313043	3	NRCAM	0.001105	0.25658	13
MCT vs. SC				PRMT5	0.000581	0.296164	17
EGFR	0.017832	0.371711	149	POLR3C	3.92E-05	0.265655	17
RPL35	5.85E-05	0.30435847	73	SEMA3A	3.9E-05	0.28154	16
COPA	0.00661	0.299735	54	TAF9B	0.00086	0.274302	15
PMF1	1.41E-05	0.309089	49	PIAS4	0.000677	0.299916	15
ARF6	0.006571	0.317375	45	THRB	0.000456	0.312586	13
FBXW11	0.002383	0.324162	44	DCAF7	0.000292	0.276992	12
SMURF2	0.003544	0.323445	40	EIF1	1.64E-05	0.240752	12
WWTR1	0.004166	0.318269	35	GSN	0.004329	0.284374	11
CACNB2	0.001283	0.313691	34	OCLN	0.001005	0.269397	11
TBL1X	0.001139	0.317051	33	AES	7.63E-05	0.308475	11
CUL3	0.001145	0.302286	28	ATP6	3.7E-07	0.186567	11
RPP30	0.000402	0.250681	28	ALDOA	0.001615	0.264891	4
AP1M2	5.86E-05	0.281063	26	HSD17B8	0.666667	1	3
TGFB2	0.002654	0.314127	25	SC vs. LC			
PHAX	0.000336	0.294655	23	NCBP1	0.153881	0.26259	17
FABP4	0.000212	0.297939	23	KDELR2	0.01875	0.533333	7
SEC23A	0.001501	0.258339	22	FOXO1	0.152968	0.264493	5
CXCL9	6.9E-05	0.269514	21	EDN1	0.121988	0.302905	7
ND2	5E-05	0.215126	21	PSMA5	0.066667	0.769231	7
CTF1	0.000322	0.267398	19	ITGB5	0.134354	0.25	5
CITED2	9.8E-05	0.289845	19	PPIE	0.105023	0.216617	7

### Pathway enrichment analysis was performed

**Fig. 3:**
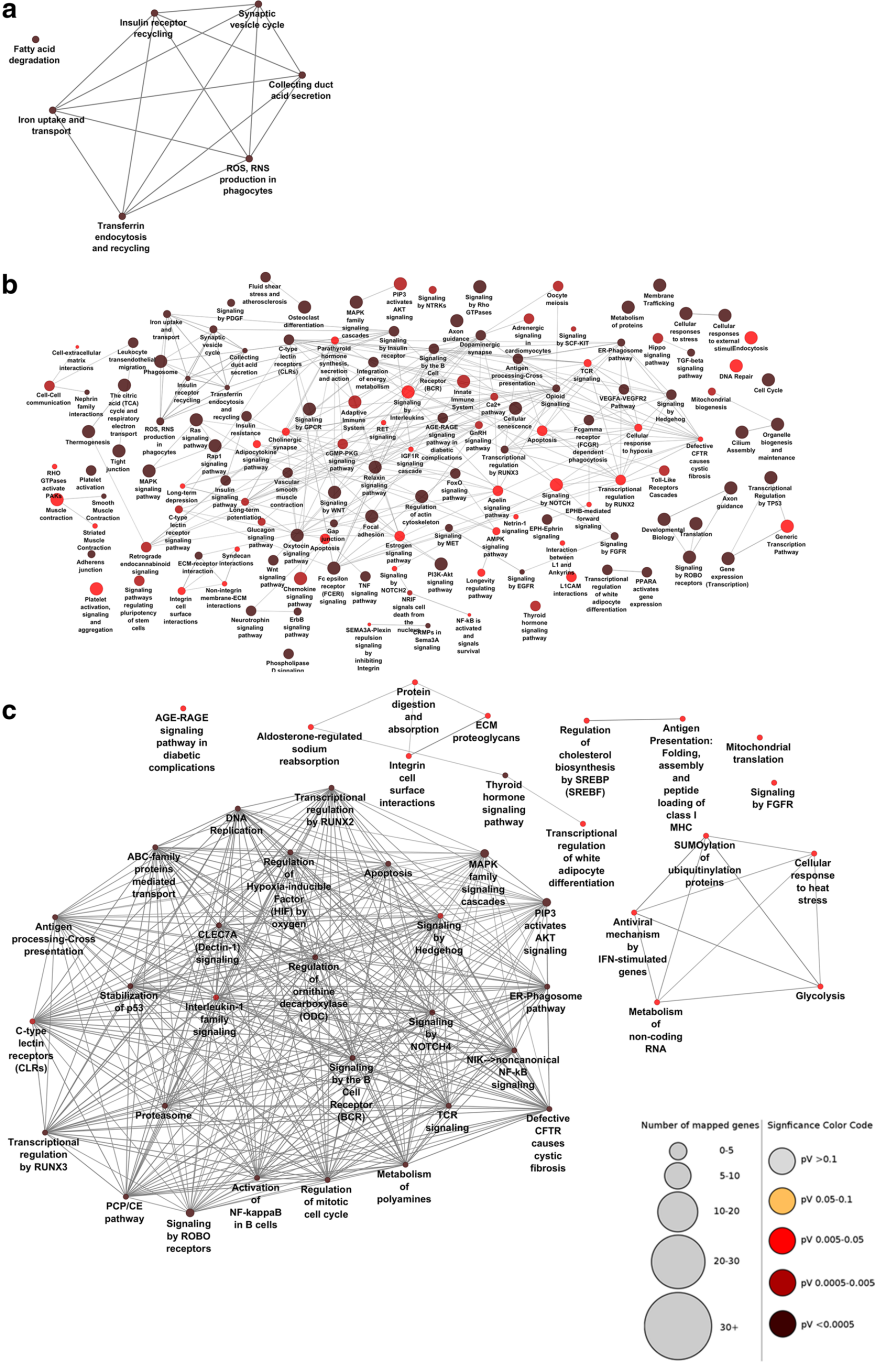
Pathway enrichment analysis of clustered genes. Functional analysis showed interconnected and informative pathways mainly are associated with renal cystic growth (**a** Normal vs. MCT, **b** MCT vs. SC, and **c** SC vs. LC). The significance of pathways is labeled based on the color code. The number of mapped genes in each path is shown according to the size of nodes

Functional analysis was carried out based on genes detected by MCODE. Using pathway enrichment analysis from 18, 1318, and 66 genes, we reached to 7, 113, and 39 pathways, respectively (Fig. 3). Interestingly, the GoTerms are informative and related to the phenotype of each step, such as collecting duct acid secretion in early step. An interesting finding in this study was the detection of critical pathways and functions such as EGF, Wnt, MAPK, HIF, P53, CFTR, AMPK, PDGF, NFκB, IGF1, MET signaling, oxidative phosphorylation, energy metabolism, cell–cell and cell–matrix interaction, and signaling by interleukins which were previously shown to be associated with ADPKD in experimental studies [19–21], and other pathways could consider for more studies and validation.

### miRNAs and TFs enriched with DE gene were determined

**Fig. 4:**
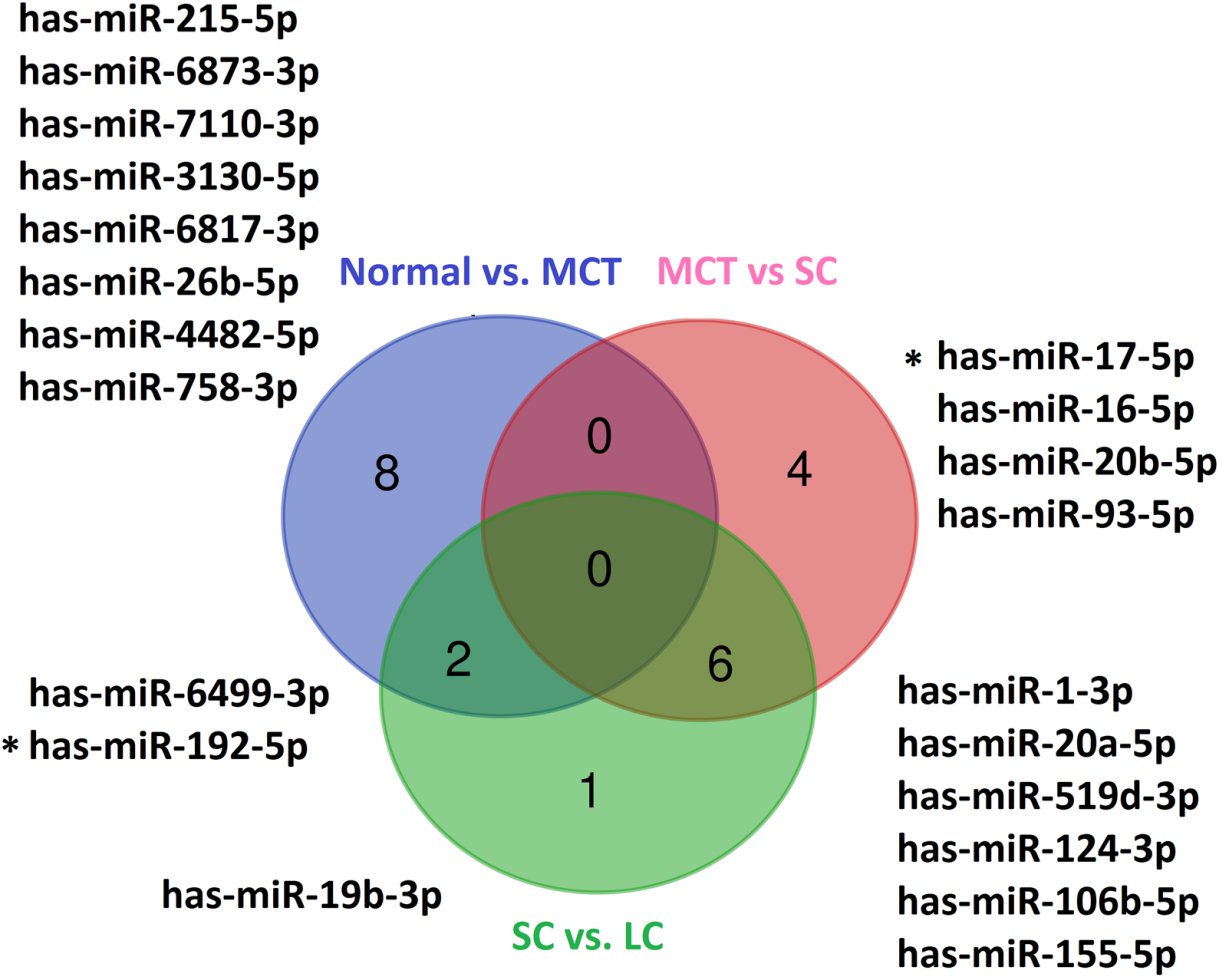
miRNA enrichment analysis results. The top of the miRNA were predicted. An adjusted p-value less than 0.05 was considered as the significant cut-off

The miRNAs and TFs as important regulators of DE genes were predicted. HNF4A, ESR1, and RXR were defined as TFs in the initial step, in normal vs. MCT comparison. TFs were significant in the small and large cyst growth steps are shown in Table 2. The top miRNAs enriched with DE genes in each phase are shown in Fig. 4. Previous studies reported the association of ADPKD with some of TFs e.g. HNF4A, STAT3, VDR, TP53, and HIF1A [6, 20, 22, 23]. Also, the role of miR-17 family and miR-192 in cyst enlargement were identified [24, 25]. It is valuable to investigate other miRNAs and TFs in experimental studies.

**Table 2:** Transcription factor enrichment analysis

Normal vs. MCT	MCT vs. SC		SC vs. LC	
HNF4A	CLOCK	ESR1	BRD4	VDR
ESR1	ZNF217	TCF21	NERF2	AR
RXR	NUCKS1	ESR2	CEBPD	PPARG
	RELA	NR1H3	TOP2B	KDM5B
	WT1	OLIG2	SMARCA4	TP53
	PAX3-FKHR	SOX9	STAT3	MYB
	CEBPD	FOXA1	TP63	KDM5A
	SMARCA4	AR	EBF1	EKLF
	FOXA2	KLF4	PAX3-FKHR	FOXA1
	PRDM5	MYB	SOX2	CEBPB
	SOX2	CTNNB1	ZNF217	PCGF2
	HNF4A	KLF6	NFE2L2	
	NFE2L2	P300	NRF2	
	NRF2	VDR	SMAD4	
	SMAD4		HNF4A	

TFs were obtained with adjusted p-value < 0.05

## Discussion

ADPKD caused by mutations in PKD1 or PKD2 genes [2] and the protein products of these genes, polycystin-1 and polycystin-2 act as a mechanosensor on the surface of epithelial and endothelial cells [4]. The loss and gain of function of these proteins, leading to dysregulation of pathways related to proliferation, apoptosis, and polarity of cells [5]. Despite many studies indicated the functions of the polycystins, the numerous ambiguities remain about the molecular mechanisms of the disease progression. For the importance of time series analysis of diseases [26], the purpose of this study was the computational analysis of the expression profile of renal cysts that were compared based on different sizes of cysts. Bioinformatics methods were performed in this study showed that 512, 7024, and 655 DE genes, respectively dysregulated in each step. The PPI networks were shown nodes and their interactions became more complex with the progression of disease in small cyst growth. The topology and clustering analysis of networks were employed for revealing candidate genes with high centrality as therapeutic targets. Nodes (genes) with high degree, they have many connections and are important for the networks. Betweenness centrality is based on the number of shortest paths going through a node and are shortcuts of the networks. Also, closeness centrality calculated physically nearest genes to all nodes [27]. Modules are high density regions in the network and identify functional genes [12]. The role of some of these genes has been well documented in ADPKD such as EDN1 as a vasoconstrictor may promote tumorigenesis and recent studies have documented that an increase in serum endothelin levels is associated with renal pathogenesis of ADPKD. Also, polymorphisms of EDN1 can influence the age of onset of end-stage renal disease in ADPKD [18, 28]. EGFR promotes cell growth, proliferation, and cell survival and has important functions in the progression of ADPKD [17]. Other genes introduced as applicant genes for future studies are ARF6, SMURF2, WWTR1, CACNB2, and FOXO1. ARF6 is a member of the RAS superfamily that regulates signaling pathways related to actin remodeling such as wnt path, the central pathway in ADPKD [26]. SMURF2 controls cell migration with BMP and TGFβ signaling pathways [29]. WWTR1 acts as a transcriptional coactivator downstream of the Hippo signaling pathway that plays a major function in the control of organ size [30]. Ablation of CACNB2 leads to calcium homeostasis derivation and could have a critical role in the initiation and progression of the disease. Previous studies showed that mutation in the PKD1 leads to higher glycolysis in ADPKD kidneys. FOXO1 through insulin signaling plays a main role in glucose metabolism and consequently involved in ADPKD pathogenesis [31, 32]. Also, ITGB5 contributes to cell adhesion and known as a biomarker in kidney disease [33]. The mechanisms of the newly introduced crucial genes such as PPIE remain to be identified with experimental studies. We pointed out TFs such as HNF4A, STAT3, VDR, TP53, and HIF1A associated with ADPKD [22, 23]. In addition, other TFs as CLOCK in ADPKD pathogenesis firstly are described in this study. Since CLOCK involved in kidney function, confirmation its role in ADPKD can get interesting results [34]. Functional analysis was shown that the pathways are correlated with the phenotype of disease in each step including pathways involved in cell proliferation, apoptosis, and inflammation. The roles of some of the pathways have determined in ADPKD pathogenesis [19, 20].

## Conclusions

Here by computational tools we generate a systematic view of the ADPKD to explore the comprehensive molecular mechanisms of a monogenic disease. Methods employed in this study may also be used for each monogenic disorder to reach novel therapeutic targets. Also, the necessity of holistic maps assay of monogenetic disease besides complex disease is desired.

## Supplementary information

**Additional file 1.**

## Data Availability

All data analyzed during this study are included in article and its additional files.

## Abbreviations

ADPKD: autosomal dominant polycystic kidney disease
PPI: protein–protein interaction
DE: differentially expressed
PKD1: polycystic kidney disease-1
PKD2: polycystic kidney disease-2
PC-1: polycystin-1
PC-2: polycystin-2
GSEA: gene set enrichment analysis
GEO: Gene Expression Omnibus
PCA: principal component analysis
FDR: false discovery rate
MCODE: Molecular Complex Detection
SC: small cyst
LC: large cyst
MCT: minimally cystic tissue
miRNA: microRNA
TF: transcription factor
KEGG: kyoto encyclopedia of genes and genomes
